# Science as a Vocation in the Era of Big Data: the Philosophy of Science behind Big Data and humanity’s Continued Part in Science

**DOI:** 10.1007/s12124-018-9447-5

**Published:** 2018-07-05

**Authors:** Henrik Skaug Sætra

**Affiliations:** Faculty of Business, Languages, and Social Science, Østfold University College, Remmen, 1757 Halden, Norway

**Keywords:** Science, Big data, Philosophy of science, Creativity, Art, Values

## Abstract

We now live in the era of big data, and according to its proponents, big data is poised to change science as we know it. Claims of having no theory and no ideology are made, and there is an assumption that the results of big data are trustworthy because it is considered free from human judgement, which is often considered inextricably linked with human *error*. These two claims lead to the idea that big data is the source of better scientific knowledge, through more objectivity, more data, and better analysis. In this paper I analyse the philosophy of science behind big data and make the claim that the death of many traditional sciences, and the human scientist, is much exaggerated. The philosophy of science of big data means that there are certain things big data does very well, and some things that it cannot do. I argue that humans will still be needed for mediating and creating theory, and for providing the legitimacy and values science needs as a normative social enterprise.

## Introduction

*This is a world where massive amounts of data and applied mathematics replace every other tool that might be brought to bear. Out with every theory of human behavior, from linguistics to sociology. Forget taxonomy, ontology, and psychology. Who knows why people do what they do? The point is they do it, and we can track and measure it with unprecedented fidelity. With enough data, the numbers speak for themselves (*Anderson 2008*).*

Many say we now live in the *era of big data* (Boyd and Crawford 2012; Chen et al. 2012; Sivarajah et al. 2016). Big data is a phenomenon revolving around the endeavour of accumulating massive amounts of data and using it to understand the objects from which we are gathering data. Combined with the technology of machine learning and artificial intelligence, it is possible to argue that much knowledge is now produced autonomously by the *tools* scientists have made, and not directly by the scientists themselves. As big data has gained prominence, it has spread from the fields of business and computer science into society at large and just about every other science. Is the crowning achievement of the era of big data that big data itself becomes some form of master science, displacing other forms of science and changing the role of the human scientist completely?

Proponents of big data often make the dual claims of having no theory and no ideology. In addition to this, there is an assumption that the results of big data are trustworthy because it is considered free from human judgement, which is often considered inextricably linked with human *error*. These two claims lead to the idea that big data is the source of better scientific knowledge, in particular with respect to objectivity. I argue that that big data *is* better at some forms of objectivity, particularly the *mechanical* and *aperspectival* versions, but that this comes at a price (Daston 1992). I show that in terms of philosophy of science big data has a certain likeness to behaviourism, and that this means that there are certain things big data *can* be used for, and other things it *cannot* help us with. My main argument is, therefore, that there will still be a role for human beings in science, both when it comes to mediating and creating theory and for providing the legitimacy and values science needs as a normative social enterprise.

## The Advent of Big Data and the Philosophy of Science behind it

Big data is a term that may simply refer to large amounts of data, in which case it would not be particularly useful. Humans have been gathering data for a long time and have become increasingly proficient at gathering larger and larger amounts of it (Marr 2015). I choose to use the term for describing today’s usage of the term, which according to Marr's (2015) brief history of big data started with Anderson's (2008) article that was quoted at the very beginning of this paper. There are, however, important similarities between the rise of big data and previous quantitative endeavours in science, and these will be commented upon where relevant. I rely on Laney's (2001) well established definition of big data, which consists of the *three V’s*: *volume*, *velocity*, and *variety* (Laney 2001). Volume refers to the massive amounts of data collected, velocity to the speed of data generation and analysis, and variety to the “structural heterogeneity” in the data (Gandomi and Haider 2014, p. 137). Big data has become an interdisciplinary phenomenon, and the amounts of data we now have means there are new requirements for both data analysis and data handling (Bello-Orgaz et al. 2016, p. 45; Boyd and Crawford 2012, p. 662).

In order to determine what role big data can and cannot play in science, it is necessary to examine its underlying philosophy of science. What assumptions are made when employing big data – implicitly and explicitly? Furthermore, what consequences does these assumptions have for the possibility of generating knowledge with the use of big data? I will first examine some of the claims of theory neutrality and the focus on behaviour. Then I will look briefly at the possibility of the computer as a scientist, followed by some critical remarks related to the neutrality, ideology and research agendas of big data. The section is concluded by a brief summary of the philosophy of big data.

### Post-Theory and Behaviorism

Brooks (2013) states that “[t]he theory of big data is to have no theory, at least about human nature. You just gather huge amounts of information, observe the patterns and estimate probabilities about how people will act in the future” (Brooks 2013). This statement implies that big data is set to become some sort of master science, making traditional disciplines obsolete, and the theoretical activity of classical science a thing of the past. The quote seems to imply that big data, by its logic of accumulation and discovery of patterns our human minds are unable to find, makes disciplines such as philosophy and parts of psychology obsolete. It is as if certain theories, for example those of human nature that has long guided the social sciences, has only been an endeavour undertaken in lieu of the possibility of gathering enough data and analysing it properly.

The ideology behind big data is in many ways similar to the approach of the behaviourists in psychology. According to this approach, the “black box” of conscious thought is either considered to be of little interest or it is simply assumed to be unavailable for scientific purposes, and thus ignored. Koestler (1967) is an interesting critic of the advent of behaviourism. He readily admits that a lot can be explained by behaviourism, but that there are obviously many things that can *not* be captured by this approach. He mentions “scientific discovery and artistic originality” as one such field, something I return to in the section where I discuss the role of the human scientist (Koestler 1967, p. 13).

What people *think* they want, and what they *think* they are like, is of little interest. The data we gather lets us know individuals better than they know themselves, so why bother with subjective perceptions? Psychology may still continue as a science of explaining what goes on inside people’s minds, and how they experience the world, but for understanding what they’ll do, theories of motivation, preferences, and actions are no longer needed when we have big data. If such a quest for an objective science of man and society accurately portrays the rise of big data, the following words by Burt, addressed at the rise of behaviourism, seems to have continued relevance today:

*The result, as a cynical onlooker might be tempted to say, is that psychology, having first bargained away its soul and then gone out of its mind, seems now, as it faces an untimely end, to have lost all consciousness (*Burt 1962*, p. 229).*

This is somewhat similar to the economic theory of *revealed preferences*. Here we infer preferences from behaviour alone. Individuals are observed, and “the individual guinea-pig, by his market behaviour, reveals his preference pattern – if there is such a consistent pattern” (Samuelson 1948, p. 243). By asking people what they want we are wasting time, because a) it is unnecessary since we have the data to predict it, and b) because people might not really know themselves or the basis of their actions anyway. This resembles what Sayer (2010, p. 22) labels *radical behaviourism*, whose “proponents insist that the meanings people attach to their actions and to other objects play no part in determining what they do”. How big data relates to the concept of liberty and identity formation are also very important issues, but something I will not pursue in this paper. For readers interested in these topics, I recommend reading Cohen (2013) and Yeung (2017).

### The Computer Scientist, as in: The Computer that Does Science

In the introduction I mentioned that some see big data as computers doing science without human involvement. Sure, we have built the computers and the tools for gathering and analysing data, but these tools now to a certain degree act without our supervision. What we mean by computer “intelligence” has changed drastically from the early days of computing. Human programmers previously made detailed instructions for the computers, detailing the possible situations that could arise (“if”-clauses) and telling the computer what the proper reaction is (“then”-instructions). What is new with machine learning is that we instead of giving detailed instructions on how to react to given situation merely give the computers certain *goals* and a *data set.* We then set the computer to work, rather blindly, and let it sift through all the material in order to find interesting patterns. For purely analytical purposes the patterns and correlations themselves are interesting. In addition to discovering patterns, we employ artificial intelligence that *acts* on this information and adjusts the various variables under its control in order to reach the given goal in the best possible way. This is the approach that lets computers beat humans at games such as Chess and Go, stealing headlines and awing professionals and amateurs alike by moves far beyond our grasp (Campbell et al. 2002; Chouard 2016; Google 2018; Kurzweil 2015, p. 148).

Theoretically blind, with human nature being considered both unnecessary for, and even an obstacle to, good science; big data analysts “are not like novelists, ministers, psychologists … coming up with intuitive narratives to explain the causal chains of why things are happening” (Brooks 2013). Neither “a priori knowledge, nor hermeneutic sensibilities” is required for gaining insight with big data, and human beings are little more than “data custodians and curators” (Baruh and Popescu 2017, p. 583). This then, is an age of “knowledge without visionaries”, with “innovation without innovators, purged of the sloppiness, bias and incompleteness that attends ordinary human endeavours” (Cohen 2013, p. 2921). Cohen points to the irony of the fact that while we hold innovation so highly we are at the same time “seeking a modality for innovation that will transcend individual agency altogether” (Cohen 2013, 1922). Chen et al. (2012) discuss the various ways in which big data will impact various fields, and in science and technology they list “S&T innovation” and “knowledge discovery” as two of the applications of big data, and the impacts of big data application will be advances in science (Chen et al. 2012, p. 1173). The authors are, however, more focused on how big data lets scientists analyse *more* data more *effectively* than on big data *changing* science.

When humans become little more than “data custodians and curators” it is easy to imagine a future in which the education of scientists consists of teaching students of various disciplines programming, data analysis, and advanced statistics. The advice for students looking to be attractive is clear: learn to handle and analyse data, learn “databases, machine learning, econometrics, statistics, visualization, and so” (Chen et al. 2012, p. 1165–6). The most successful up-and-comers “no longer work for “Enron, Lehman Brothers, or AIG; now they work for Goole or Target or Acxiom, pursuing the holy grail of knowing customers better than they know themselves” (Cohen 2013, p. 1923). The fascination with statistics and its potential is, however, not new. In 1897 Oliver Wendell Holmen Jr. stated that “the man of the future is the man of statistics” (Cohen 2013, p. 1928). Some also point to the similarities between big data and previous quantitative movements in science. One of these was geography’s quantitative revolution some 45 years ago, which lead to many of the same discussions that we are having in relation to the rise of big data (Barnes 2013). One central point in these quantitative shifts is that when theory creation is no longer a business for humans, our role becomes limited to making the systems that do science and setting them to work. I will return to the obvious point that choosing where and how to employ these systems is still a very human aspect of big data.

### Ideology and Research Agendas

There are three fundamental problems with big data, according to Cohen (2013). First of all is the fact that the research agendas are often hidden, and “observers have begun to point to a ‘credibility crisis’ that derives from inadequate disclosure of data sets and methods” (Cohen 2013, p. 1924). This is related the point made by Gandomi and Haider (2014) that big data has bypassed academic analysis. Much of what is done with big data is done by large corporations that do not publish in academic channels or adhere to academic standards of transparency and disclosure. Businesses adopting various methods to achieve their own goals without disclosing them is of course no new phenomenon. However, the fact that big data has become so powerful, and that so much of it is controlled by corporations, makes it a more pressing issue – especially relating to issues of power dynamics, individual liberty and the formation of selves (Cohen 2013). An issue related to this problem is that machine learning based on big data sometimes *fail*. Due to the complexity of big data analytics, humans have a hard time understanding the results of machine learning. This is quite natural, due to the fact that these computer systems have surpassed human capacities in many areas. A thorough examination of how and why big data analytics sometimes fails is beyond the scope of this paper, but some examples shows the important of the issue. First of all, big data has “marginalized regulatory schema” by “evading current privacy protections with its novel methodology” (Crawford and Schultz 2014). This is a problem, the authors state, because the predictive use of big data profiles today influences people’s lives and opportunities in many important ways, but the profiles may be deeply problematic (Crawford and Schultz 2014). They may simply be *inaccurate*, in which case the effects are varied and hard to evaluate, but they can also be discriminatory in several ways. First of all, analysis based purely on historical data will contain many traces of historical discriminatory practices, which cannot be corrected simply by removing variables such as gender and ethnicity. Furthermore, companies can actively circumvent anti-discrimination policy by finding proxies for the variables that they are forbidden to base their choices on (Crawford and Schultz 2014, p. 100). Even if you remove *gender* from a data set, there are countless ways to identify women by looking at the vast amounts of other variables and data available, and thus veiling discriminatory practices. Hirsch (2014) is another good source for more information on how big data can lead to both intended and accidental discrimination practices.

The second is that there is an underlying ideology inherent in big data, as it “is the ultimate expression of a mode of rationality that equates information with truth and more information with more truth, and that denies the possibility that information processing designed simply to identify ‘patterns’ might be systematically infused with a particular ideology” (Cohen 2013, p. 1924). Big data is not, and cannot be, neutral, and the concept of *predictive rationality* is fundamental to the phenomenon. Predictive rationality is most often used in management literature and is sometimes uses as a synonym to *rational choice theory* (Flowers et al. 2017). According to Sarasvathy (2001) it involves a) goals, b) alternative means or causes to the goal, c) constraints on means, and d) criteria for selecting between means (Sarasvathy 2001, p. 249). The problem, however, is that the ideology and the values it endorses are hidden, and often pretended away (Cohen 2013, p. 1925). There are several strands in this argument: First of all, we have the fact that human beings build the systems that interpret big data, they are involved in tweaking these systems, in where they are applied, and finally in interpreting how they work. This human involvement is at times veiled, or pretended away, but it is hard to argue that no ideology can be introduced in any (or all) of these stages. The second, and vitally important, part, is that Anderson's (2008) view that “[w]ith enough data, the numbers speak for themselves” is what Barnes (2013, p. 300) calls “data determinism with a vengeance”. The idea that data can be considered neutral is a view that has been attacked by many, but in this context, it suffices to relate Sayer's (2010) important criticism of this position. He states that “theory is increasingly recognized as affecting observation itself, so that the latter is said to be ‘theory-laden’“ (Sayer 2010, p. 46). Barnes (2013, p. 300) nicely summarises the main points of Sayer’s criticism of the uncritical use of quantification and observation, in that “numbers are never innocent, speaking for themselves, but always come marked by prior theorization: they are theory laden” and “emerge only from particular social institutions, arrangements and organizations mobilised by power, political agendas and vested interests”. Knowledge based solely on experience and observation “then becomes at least highly ambiguous” (Sayer 2010, p. 46). Lastly, we have the fact that big data, as a purely descriptive science, “is inherently conservative” (Barnes 2013). As a descriptive science, big data must assume that the future will resemble the past, or at the very least that past trends will develop in similar ways into the future. When my predictions, recommendations and understandings are wholly based on past data, this necessarily leads towards a preservation of what is – the status quo. While not a bad thing in and of itself, it must be understood, and some will desire theories that does not “merely describe the world, that is, simply conformed to the data, but changed the world, and along with it the numbers itself” (Barnes 2013, p. 300).

The third problem is that big data leads to a society in which subjectivity is shaped “in the service of self-interested agendas of powerful economic actors” (Cohen 2013, p. 1925). This is a very important problem, but one that cannot be examined in more detail in the current paper. Subjectivity is itself subject to influences, and the problem arises when the “techniques of Big Data subject individuals to predictive judgements about their preferences, and the process of modulation also shapes and produces those preferences” (Cohen 2013, p. 1925). In short: there is little transparency and control, there is an underlying ideology that is often ignored, and the techniques employed has real effects of individuals and society.

Baruh and Popescu (2017) refers to Cohen (2013) and her suggestion that “the ideological effect of big data is the denial of the existence of ideology and bias” (Baruh and Popescu 2017, p. 583). Boyd and Crawford (2012) has written an article with 6 challenges to big data, whereas the two first is that big data “changes the definition of knowledge” and that its “[c]laims to objectivity are misleading” (Boyd and Crawford 2012, p. 662).

When proponents of big data suggest that their approach is neutral, and that one has transgressed the traditional obstacles to an objective science, that cannot be taken as much more than a lack of understanding of how science and methodology works. In the words of Cohen (2013), “the denial of ideology is itself an ideological position” (Cohen 2013, p. 1924).

### Practical Philosophical and Methodological Choices (or Necessities)

It is time now to summarise some of the above and position big data in relation to some of the central concepts used in science and methodology. In terms of philosophy of science, big data can surely be described as a positivist scheme. Positivism is in textbooks on research methods characterised by *phenomenalism, deductivism, inductivism, objectivity*, and science as a *positive/descriptive,* and not normative, endeavour (Bryman 2008, p. 13). I’ll return to the point of inductivism, and will only note that the reason both deduction and induction is mentioned, comes from the fact that we do create theories from the data we gather, and we also gather the data we do because we are to a certain degree guided by theories (Bryman 2008, p. 13).

It is clearly empiricist, in that the only acknowledged source of knowledge is observable facts, mediated by reason and logic. The observable facts are the data we gather in various ways, whereas machine learning and AI is used to supply the reasoning and the logic necessary to create knowledge from the data. Related to this is big data’s logic of aggregation, which relates to Cohen’s description of this approach as one “that equates information with truth and more information with more truth” (Cohen 2013, p. 1924). With big data and machine learning, more information both strengthens the resulting theories, *and* increases the capacities of our computers through the improved learning opportunities. However, if observations are theory-laden, it is difficult to accept a position that considers it a foundation of truth without the need of mediation (Sayer 2010).

Theories about correlations and causation can thus be formed a posteriori by smart computers capable of spotting patterns and analysing how variables are connected and what variables causes change in others. There is no need for human activity in this process, as the parts required by humans has already been played out when we provided the machines with knowledge of statistics and what qualifies as experience in the form of data sets, plus the ability to learn without our help just by going through these data sets. As such, big data is an **inductive** scientific enterprise, gathering and the going through it in order to find patterns and build theories about dependencies and causation. However, if we look at big data science where computers are involved in explaining the findings and making sense of the many possible hypothesis put forth by computers, we have science that resemble “a type of reasoning that begins by examining data and after scrutiny of these data, entertains all possible explanations for the observed data, and then forms hypotheses to confirm or disconfirm until the researcher arrives at the most plausible interpretation of the observed data” (Charmaz 2006, p. 186). This is Charmaz' (2006) definition of *abductive* science, and Kitchin (2014, p. 5) states that big data science is rather pragmatic, and open for “hybrid combination of abductive, inductive and deductive approaches to advance the understanding of a phenomenon”.

Big data is surely quantitative in its nature, and “the ideology of big data naturalizes algorithmic analysis of quantitative data as the paramount expression of truth” (Baruh and Popescu 2017, p. 583). Proponents of big data may argue that you could also analyse qualitative material like texts, music and photographs by method of big data, but that would imply a lack of understanding of how computers actually analyse the material. Yes, the mentioned forms of data may be considered qualitative if interpreted by a human social scientist employing hermeneutics or other similar methods, but that is not what the computer does. When the computer receives a picture, it is transformed into code – into 0’s and 1’s. This is what it analyses. When working with full texts, it also employs the qualitative method of counting words, comparing structures etc., and not interpretative methods. Furthermore, as already touched upon, most of the data is coded by humans, and cannot be considered objective or neutral in themselves (Sayer 2010). Some might argue that human cognition involves decoding all impressions in similar ways, and that we just *believe* we perceive wholes and truly qualitative material. Gestalt psychology is an interesting theoretical direction in this respect, and I will assume that humans do perceive wholes as “different from the sum of its parts” (Rock and Palmer 1990).

I have also argued that big data is mainly concerned with behaviour, as opposed to cognition. I base my view on the fact that while one can surely use big data on surveys where people are asked about their evaluations or subjective experiences, the goal is not primarily to understand what goes on inside people’s minds, but to understand how these answers translates into behaviour. We may come to understand that being dissatisfied with the level of care in a municipality is connected with a desire to relocate, but big data cannot help us with the *process* that explains this correlation.

The lack of understanding of process does not mean that big data is not combined with theories of process in order to guide behaviour. A prime example is how companies use personal data from social media and combine it with psychological theories of motivation and action in order to influence voting behaviour etc. Cambridge Analytica in the 2018 scandal involving Facebook is one example (Greenfield 2018). This approach to the application of big data relies on a methodology resembling that of Sunstein and Thaler's (2003) *nudge* theory.

All of this comes together to form a picture where much of the traditional social science methodology is a poor fit, whereas natural science methodology, mathematics, econometrics, etc. fits very well. Objectivity seems to be one of the main arguments for the style of science that follows the approach of big data. We can speak of *ontological, mechanical,* and *aperspectival* objectivity, and the two last ones are perhaps particularly interesting here (Daston 1992). Aperspectival objectivity involves “eliminating individual (or occasionally group, as in the case of national styles or anthropomorphism) idiosyncrasies”, while mechanical objectivity “is about suppressing the universal human propensity to judge and to aestheticize” (Daston 1992, p. 599). When we remove the human scientist from the equation, both human propensities and human perspectives are overcome, right? One could argue that both is built into our computers, our methods of analysis, and the data itself, but for our current purposes we will be satisfied with stating that proponents of big data *claim* to achieve high scores in these layers of objectivity.

I expect some to argue that I am now shoehorning what is a relatively infantile phenomenon, filled with variation and theoretically not very well developed, into old and ill-fitting categories. This objection can hardly be dismissed, as there are surely many people employing big data in ways at odds with what I have just described. This is partly a result of the fact that much research based on big data seems rather pragmatic. Gandomi and Haider (2014) discusses this phenomenon, and the fact that big data is philosophically underdeveloped. The meteoric rise of big data has lead researchers to “leapfrog to books and other electronic media for immediate and wide circulation of their work”, instead of developing the approach in regular academic channels (Gandomi and Haider 2014, p. 137). Regardless of the somewhat eclectic philosophical approach of big data applications, I stand by my argument that these are the underlying philosophical assumptions most descriptive of the endeavour of big data. For a more detailed discussion of the philosophy of big data, see Melanie Swan’s article dedicated to the subject (Swan 2015).

## The Role for the Human Scientist

Having examined the philosophy of science of big data, it is time for humanity to make its case for still playing a part in future science. In the first part, I relate some arguments from classical philosophers of science, that together form the impression that science is a form of *art* – something not *merely* technical, but in need of both passion and creativity. I am aware that if pushed too far, this line of reasoning stands in danger of making science something *mystical.* While thus placing it outside the reach of computers, some will say I have merely replaced one problem with an even bigger problem. I do *not* claim that science is a mystical art, forever beyond the reach of computers, but I do argue that science requires certain forms of creativity and serendipitous human ingenuity that are, *at present*, not available for computers. I then relate some ideas about objectivity and normativity in science in the last part of the section.

### The Scientist as Artist

For Michael Polanyi, scientific discovery “reveals new knowledge”, but the accompanying vision is to him not knowledge; “[i]t is *less* than knowledge, for it is a guess; but it is *more* than knowledge, for it is a foreknowledge of things unknown and at present perhaps inconceivable” (Polanyi 1962, p. 135). Even further, he claims that any “process of enquiry unguided by intellectual passions would inevitably spread out into a desert of trivialities” (Polanyi 1962, p. 135).

Intellectual passions are central, as they can “evoke intimations of specific discoveries and sustain their persistent pursuit” (Polanyi 1962, p. 151). What he calls the “heuristic function” of scientific passion is that an appreciation for science merges “into the capacity for discovering it; even as the artist’s sensibility merges into his creative powers” (Polanyi 1962, p. 151). He says there is such a thing as “creative scientists” that are occupied with “trying to guess right”, and the work is creative both because of the process of discovering new knowledge and because it irreversibly changes our societies (Polanyi 1962, p. 151). When something new is discovered, new interpretative frameworks are required, as the old ones no longer help us understand what we now know. Science, in the form of discovery, is thus creative, as “it is not to be achieved by the diligent performance of any previously known and specifiable procedure” (Polanyi 1962, p. 151).

Karl Popper, in a somewhat similar vein, compares the scientist to the artist (Popper 1989). One of his main points is the somewhat disinterested (in terms of applications) way true artists and scientists pursue their creative work – “[n]either Planck nor Einstein, neither Rutherford nor Bohr, thought of a possible application of the atomic theory” (Popper 1989, p. 38). They “search for the sake of the search”, and the pursuit of science has its roots in “poetical and religious myths” and has less to do with the mere collection of facts in order to find new applications (Popper 1989, p. 39). For Popper, the aim of science is the search for truth, and the process that gets us there is an artistic one (Popper 1989, p. 40). Nowhere does he say that information alone will get us there. The picture Popper paints fits rather poorly with the idea that machines sifting through masses of data can replace the human scientist. Unless we grant computer the gift of creativity and purposeful action, that is. In this article I will assume that computers are not yet autonomous in this way. It is of course a topic of great interest, and should computers get there, it certainly has implications for the need of humans in science.

*Nowadays in circles of youth there is a widespread notion that science has become a problem in calculation, fabricated in laboratories or statistical filing just as 'in a factory,' a calculation involving only the cool and not one's 'heart and soul' (*Weber 1958*, p. 113)*

Weber, in his *Science as a Vocation*, discusses the move towards calculation and statistics in certain disciplines, and emphatically states that there must exist *ideas* in someone’s minds for the calculating endeavour to be valuable, and that “such intuition cannot be forced” (Weber 1958, p. 113). Calculation and information alone will not get us far. These *ideas* are both fickle and impossible to willingly produce, and once again we are lead into rather mystical terrain where *intuition* and *inspiration* makes science more akin to art than a purely technical process. Weber states that “inspiration plays no less a role in science than it does in the realm of art”, and while the processes of painters and scientists may be somewhat different, “the psychological processes do not differ. Both are frenzy (in the sense of Plato’s ‘mania’) and ‘inspiration’“ (Weber 1958, p. 113).

If science is an art, then, perhaps artificial intelligence is not yet quite up to the task of fully replacing the human scientist. According to Boden, “[c]reativity is a fundamental feature of human intelligence, and an inescapable challenge for AI” (Boden 1998, p. 347). She writes that there are three types of creativity, in that it either consist of a) combining known concepts in unknown ways, b) exploring the adjustment of the constellation of existing structures or c) transforming the space in which known structures exist (Boden 1998, p. 348). Although many philosophers have claimed that no such thing as a truly *new* idea arises from human creativity, we might do well not to conclude that this means that machines have the same potential for creativity. Let’s say that the combinatorial mode of creativity is what interests us. A computer may create a fantastical amount of new possible constellations of existing concepts and phenomenon, but the real task of innovation is to determine which ones have potential and which ones don’t. Here we return to the educated intuition of human beings. To the scientists with the intellectual passions Polanyi described, some new constellations will stand out and be perceived as very interesting. The scientist may not even understand why he gets this sensation, but the intuitive recognition of valuable information, derived from an education is science, is hard to program into a computer. Boden discusses the problem of giving a computer the tools needed to properly evaluate the new ideas it comes up with:

*Identifying the criteria we use in our evaluations is hard enough. Justifying, or even (causally) explaining, our reliance on those criteria is more difficult still. For example, just why we like or dislike something will often have a lot to do with motivational and emotional factors – considerations about which current Al has almost nothing to say (*Boden 1998*, p. 347).*

Boden (1998) writes that the ability of AI to both find novel ideas and convince us of its value would be the “ultimate vindication of AI”, but ends with stating that “[w]e are a very long way from that” (Boden 1998, p. 355). In a more recent article, Lake et al. (2017) discuss how new AI systems based on “deep neural networks” are inspired by human biology. Despite the progress made, “these systems differ from human intelligence in crucial ways”, and the authors specifically propose AI systems that “build causal models of the world that support explanation and understanding, rather than merely solving pattern recognition problems” (Lake et al. 2017). One of the problems computer engineers face are, as we have seen, that some “human cognitive abilities remain difficult to understand computationally, including creativity, common sense, and general-purpose rea- soning” (Lake et al. 2017, p. 3). They state that “[c]reativity is often thought to be a pinnacle of human intelligence”, and that “we are still far from developing AI systems that can tackle these types of tasks,” although the authors have hope for some progress (Lake et al. 2017, p. 24).

Even if we *should* wish to grant computers the possibility of judging (and thus perhaps creating) truly inspired works of art, a final objection to the computer scientist must be raised: science is in part a *social* and *democratic* endeavour, subject to the normative evaluations of the societies in question. What we value and desire will change over time and between cultures. The role science has in social development and in reflecting societal values can hardly be overestimated, and I am inclined to believe that this aspect of science means that there *must* be humans involved in the scientific endeavour in order for it to be perceived as both legitimate and valuable.

We have seen that Brooks (2013) described big data analysts as being “not like novelists, ministers, psychologists … coming up with intuitive narratives to explain the causal chains of why things are happening” (Brooks 2013). If this is so, big data analysts are perhaps doing something apart from science as discussed by Polanyi, Popper and Weber?

### The Objective Scientist and Science as a Normative Endeavour

Cohen (2013) brought forth some problematic aspects of big data. Firstly, the methodological choices one makes has important ramifications for the kind of answers one can get. Secondly, and perhaps most importantly, that no approach is *neutral*. The *techniques* of big data “cannot themselves decide which questions to investigate, cannot instruct us how to place data flows and patterns in larger conceptual or normative perspective, and cannot tell us whether and when it might be fair and just to limit data processing in the service of other values” (Cohen 2013, p. 1922). Human beings are still involved in building, tweaking, evaluating, applying, and interpreting the results of the computer systems used in big data, which makes statements about human obsolescence seem wildly premature. Cohen (2013) argues that big data can never “replace either human-driven modelling or the prior decisions about direction and scope that set the substantive and ethical parameters for particular programs of investigation” (Cohen 2013, p. 1923). While it cannot replace it, it certainly can *dis*place it (Cohen 2013, p. 1926). Predictive rationality can be a threat to important social values, as it can “crowd out other kinds of motivators – altruism, empathy, and so on – that might spur innovation in different directions, and it can even displace alternative “kinds of agendas for human flourishing” (Cohen 2013, p. 1926–7). As we have seen, the descriptive character of big data means that predictions and research based on it tend to protect the status quo (Barnes 2013, p. 300).

All of Cohen’s points take us back to my point about science being both a social and a democratic undertaking. Merton (1973) writes of the various ways in which science is connected to society, and that it is dependent “on particular types of social structure” (Merton 1973, p. 267). Scientists are “an integral element of society” with the obligations and interests that comes with this, and science is not a “self-validating enterprise which was in society but not of it” (Merton 1973, p. 267–8). In this context he describes the *ethos of science*, which contains the “values and norms which is held to be binding on the man of science” – norms promulgated through “prescriptions, proscriptions, preferences, and permissions” (Merton 1973, p. 268–9). While one could argue that this ethos is technical, and could possibly be implemented in autonomous computers, Merton states that these are *moral* mores that are binding due to being considered “right and good” (Merton 1973, p. 270). As of yet I know of few that are willing to consider computers capable of morality in a full sense, and not merely ethics as behaviour according to set rules.

When I call science a democratic undertaking, I am more concerned with the aspect of science that involves the power to influence one’s own society through scientific activity, or the critique of such activity. I am not stating that science is a phenomenon of democratic regimes, a point also made by Merton (1973, p. 269). However, I argue that a part of what gives science legitimacy is the democratic qualities inherent in Merton’s four “institutional imperatives” of science: universalism, communism, disinterestedness, and organised scepticism (Merton 1973, p. 270).

Another point made by Cohen is that big data creates some challenges related to research ethics. This arises from the fact that private commercial entities are in charge of both gathering and analysing much of what we understand as big data, and that this “represents the de facto privatization of human subject research, without the procedural and ethical safeguards that traditionally have been required” (Cohen 2013, p. 1925). This is obviously a big problem with how big data is employed today, but it is *not* an integral feature of big data. Big data and the underlying philosophy that I discuss in this paper could just as well follow regular standards of research ethics, including communalism as discussed by Merton (1973, p. 273).

## Conclusion

In this paper we have seen that certain forms of science are being colonised by big data. For good reason, too, when it comes to some of the science that is performed. I have not argued that big data has no place in science, but merely that there is still room for something *other* than big data and the advent of the computer as a scientist.

Some might claim that some sciences that rely on quantitative methods and the approach of the natural sciences is a good fit for big data-based science. Here they use quantification, as much information as possible, and advanced statistical methods, so all will be better with computers, they might say. Even further, the last obstacle to real objectivity, the human being, could even be replaced by computers. I have shown that while the first statements might very well be true, we are far away from being able to accept the third.

In addition, if we look at the disciplines *not* ruled by the logic of aggregation, computers have less to contribute. I compared big data science with behaviourism – even *radical* behaviourism – and the main point was that while this approach is good for certain purposes, there are things it *cannot* do. While big data analysts may explain behaviour and other easily observable and codifiable phenomenon, some of us will still be interested in the internal processes that causes behaviour. We do not get this from data, but from the softer sciences dealing with such phenomena as human motivation, cognition, philosophy and morality. This means that we still need sciences that are not in accordance with the ideals of big data and positivism. When we need interpretation instead of statistical analysis, humans do things computers can’t. When truly qualitative phenomena are to be analysed as wholes, the computers must resign. When my thoughts, and not my actions, are the goals of explanation, I must turn to humans, and when processes and causal explanations are required, I need more than observable behaviour and facts – the only things the computer accepts.

Human scientists are still necessary, for two reasons. The first is the fact that science is in many ways like the arts, as described by Polanyi, Weber and Popper. As of yet, computers can perform just about every act conceivable, but the creativity, intuition and instincts involved in the process of developing theories and guiding their applications, is still beyond reach of the computer. If we are to avoid the “desert of trivialities” that Polanyi spoke of, we need the intellectual passion of human beings. We have good reason for applying our new computer tools to scientific problems, but we also have very good reason to reject statements that theory is dead, that science is now neutral, and that there is no longer any need for humans in other roles than in creating and running computer programs.

The second reason is that the *how, what* and *why* of the scientific endeavour is not yet possible without human involvement, especially if we are to retain legitimacy and respect for the democratic basis of science as a part of a society’s pool of common goods. One aspect of this is that when some pretend that humans are not involved in big data science, they simply veil the influence some people and organisations have in the creation, application, and interpretation of the data systems and the science that results from them. Science is of course not *directed* by popular control or politics, but it certainly develops in symbiosis with society at large. Science is more than a technical exercise. It is also a *moral* undertaking, that requires it to be directed and controlled by moral beings. For now, that means that human beings still have a crucial part to play in the future of all sciences.

## References

[CR1] Anderson C (2008). The end of theory: The data deluge makes the scientific method obsolete. Wired magazine.

[CR2] Barnes TJ (2013). Big data, little history. Dialogues in Human Geography.

[CR3] Baruh L, Popescu M (2017). Big data analytics and the limits of privacy self-management. New Media & Society.

[CR4] Bello-Orgaz G, Jung JJ, Camacho D (2016). Social big data: Recent achievements and new challenges. Information Fusion.

[CR5] Boden MA (1998). Creativity and artificial intelligence. Artificial Intelligence.

[CR6] Boyd D, Crawford K (2012). Critical questions for big data: Provocations for a cultural, technological, and scholarly phenomenon. Information, Communication & Society.

[CR7] Brooks D (2013) What you’ll do next. *The New York Times*. Retrieved from http://www.nytimes.com/2013/04/16/opinion/brooks-what-youll-do-next.html

[CR8] Bryman A (2008). Social research methods.

[CR9] Burt C (1962). The concept of consciousness. British Journal of Psychology.

[CR10] Campbell M, Hoane AJ, Hsu FH (2002). Deep blue. Artificial Intelligence.

[CR11] Charmaz K (2006). Constructing grounded theory: A practical guide through qualitative analysis.

[CR12] Chen, Chiang, Storey (2012). Business Intelligence and Analytics: From Big Data to Big Impact. MIS Quarterly.

[CR13] Chouard, T. (2016). The go files: AI computer wraps up 4–1 victory against human champion. *Nature News*.

[CR14] Cohen JE (2013). What privacy is for. Harvard Law Review.

[CR15] Crawford K, Schultz J (2014). Big data and due process: Toward a framework to redress predictive privacy harms. BCL Rev.

[CR16] Daston L (1992). Objectivity and the escape from perspective. Social Studies of Science.

[CR17] Flowers, S., Meyer, M., & Kuusisto, J. (2017). Capturing the innovation opportunity space: Creating business models with new forms of innovation. Edward Elgar Publishing.

[CR18] Gandomi A, Haider M (2014). Beyond the hype: Big data concepts, methods, and analytics. International Journal of Information Management.

[CR19] Google. (2018). Solve intelligence. Use it to make the world a better place*.* Retrieved from https://deepmind.com/about/

[CR20] Greenfield, P. (2018, March 26^th^). The Cambridge Analytica files: The story so far. The Guardian. Retrieved from https://www.theguardian.com/news/2018/mar/26/the-cambridge-analytica-files-the-story-so-far

[CR21] Hirsch, D. D. (2014). That's unfair-or is it: Big data, discrimination and the FTC's unfairness authority. Ky. LJ, 103, 345.

[CR22] Kitchin R (2014). Big data, new epistemologies and paradigm shifts. Big Data & Society.

[CR23] Koestler A (1967). The ghost in the machine.

[CR24] Kurzweil R, Schneider S (2015). Superintelligence and singularity. Science fiction and philosophy: From time travel to superintelligence.

[CR25] Lake, B. M., Ullman, T. D., Tenenbaum, J. B., & Gershman, S. J. (2017). Building machines that learn and think like people. *Behavioral and Brain Sciences, 40*. 10.1017/S0140525X16001837 27881212

[CR26] Laney, D. (2001). 3D data management: Controlling data volume, velocity and variety*. META Group Research Note, 6*(70).

[CR27] Marr, B. (2015). A brief history of big data everyone should read. In *World Economic forum blog*. Retrieved from https://www.weforum.org/agenda/2015/02/a-brief-history-of-big-data-everyone-should-read/

[CR28] Merton RK (1973). The sociology of science: Theoretical and empirical investigations.

[CR29] Polanyi M (1962). Personal knowledge: Towards a post-critical philosophy.

[CR30] Popper K (1989). Creative self-criticism in science and in art. Diogenes.

[CR31] Rock I, Palmer S (1990). The legacy of gestalt psychology. Scientific American.

[CR32] Samuelson PA (1948). Consumption theory in terms of revealed preference. Economica.

[CR33] Sarasvathy SD (2001). Causation and effectuation: Toward a theoretical shift from economic inevitability to entrepreneurial contingency. Academy of Management Review.

[CR34] Sayer A (2010). Method in social science: A realist approach.

[CR35] Sivarajah U, Kamal MM, Irani Z, Weerakkody V (2016). Critical analysis of big data challenges and analytical methods. Journal of Business Research.

[CR36] Sunstein Cass R., Thaler Richard H. (2003). Libertarian Paternalism Is Not an Oxymoron. The University of Chicago Law Review.

[CR37] Swan, M. (2015, March). Philosophy of big data: Expanding the human-data relation with big data science services. In *Big Data Computing Service and Applications (BigDataService), 2015 IEEE First International Conference on* (pp. 468-477). IEEE.

[CR38] Weber M (1958). Science as a vocation. Daedalus.

[CR39] Yeung K (2017). ‘Hypernudge’: Big data as a mode of regulation by design. Information, Communication & Society.

